# The effect of liraglutide on renal function in type 2 diabetes: a meta-analysis of randomized controlled studies

**DOI:** 10.4314/ahs.v22i3.28

**Published:** 2022-09

**Authors:** Cheng Luo, Dongjuan He, HongBin Yang, Chunyan Zhu, Jiajun Zhu, Zhaohui Hu

**Affiliations:** Department of Endocrinology, The Quzhou Affiliated of Wenzhou Medical University, Quzhou People's Hospital, Zhejiang, P.R. China

**Keywords:** liraglutide, renal function, type 2 diabetes, randomized controlled trials

## Abstract

**Introduction:**

The efficacy of liraglutide on renal function in type 2 diabetes remains controversial. We conduct a systematic review and meta-analysis to explore the influence of liraglutide versus placebo on renal function in type 2 diabetes.

**Methods:**

We search PubMed, EMbase, Web of science, EBSCO, and Cochrane library databases through March 2020 for randomized controlled trials (RCTs) assessing the effect of liraglutide versus placebo on renal function in type 2 diabetes. This meta-analysis is performed using the random-effect model.

**Results:**

Seven RCTs are included in the meta-analysis. Overall, compared with control group in type 2 diabetes, liraglutide treatment shows no obvious effect on GFR (SMD=0.02; 95% CI=-0.43 to 0.47; P=0.94), RBF (SMD=-0.28; 95% CI=-0.80 to 0.24; P=0.29) or death (RR=1.93; 95% CI=0.71 to 5.21; P=0.20), but is associated with significantly decreased ACR (SMD=-0.82; 95% CI=-1.39 to -0.26; P=0.004) and systolic blood pressure (MD=-9.60; 95% CI=-17.46 to -1.73; P=0.02), as well as increased heart rate (MD=5.39; 95% CI=3.26 to 7.52; P<0.00001).

**Conclusions:**

Liraglutide treatment may provide some benefits for protecting renal function in type 2 diabetes.

## Introduction

As the increase in diabetes pandemic, diabetic kidney disease has emerged as the leading cause of chronic kidney disease, which may cause end-stage kidney disease, cardiovascular events, and premature death^1^–^4^. Early detection of albuminuria and decline of glomerular filtration rate (GFR) help to treat diabetic kidney disease^5^, ^6^. It is also important to control renal risk factors such as hyperglycemia, obesity, systemic hypertension, glomerular hyper filtration, albuminuria, and dislipidemia^1^.

Glucagon-like peptide 1 (GLP-1)-based therapies including dipeptidyl peptidase-4 inhibitors (DPP-4Is) and GLP-1 receptor agonists (GLP-1Ras such as liraglutide), have been widely used for type 2 diabetes by improving pancreatic islet cell function, and reducing glucagon secretion^7^–^10^. In experimental models of diabetes and hypertension, GLP-1-based therapies was documented to prevent the onset and progression of renal disease, renal morphological abnormalities of diabetic kidney disease^11^.

Recently, several studies have investigated the efficacy of liraglutide on renal function for type 2 diabetes, but the results are conflicting^12^–^15^. This systematic review and meta-analysis of RCTs aims to assess the impact of liraglutide versus placebo on the renal function in patients with type 2 diabetes.

## Materials and methods

This systematic review and meta-analysis are performed based on the guidance of the Preferred Reporting Items for Systematic Reviews and Meta-analysis statement and Cochrane Handbook for Systematic Reviews of Interventions^16^,^17^. No ethical approval and patient consent are required because all analyses are based on previous published studies.

### Literature search and selection criteria

We systematically search several databases including PubMed, EMbase, Web of science, EBSCO, and the Cochrane library from inception to March 2020 with the following keywords: “liraglutide”, and “diabetes”, and “renal function” or “kidney function”. The reference lists of retrieved studies and relevant reviews are also hand-searched and the process above is performed repeatedly in order to include additional eligible studies.

The inclusion criteria are presented as follows: (1) study design is RCT, (2) patients are diagnosed with type 2 diabetes, (3) intervention treatments are liraglutide versus placebo, and (4) outcomes should involve the effect on renal outcomes.

### Data extraction and outcome measures

Some baseline information is extracted from the original studies, and they include first author, number of patients, age, female, body mass index, duration of diabetes, and detail methods in two groups. Data are extracted independently by two investigators, and discrepancies are resolved by consensus. We have contacted the corresponding author to obtain the data when necessary.

The primary outcomes are glomerular filtration rate (GFR) and renal blood flow (RBF). Secondary outcomes include albumin-to-creatinine ratio (ACR), systolic blood pressure, diastolic blood pressure, heart rate and death.

### Quality assessment in individual studies

The methodological quality of each RCT is assessed by the Jadad Scale which consists of three evaluation elements: randomization (0–2 points), blinding (0–2 points), dropouts and withdrawals (0–1 points)^18^. One point would be allocated to each element if they have been conducted and mentioned appropriately in the original article. The score of Jadad Scale varies from 0 to 5 points. An article with Jadad score≤2 is considered to be of low quality. The study is thought to be of high quality if Jadad score≥3^19^.

### Statistical analysis

We assess mean difference (MD) or standard mean difference (SMD) with 95% confidence interval (CI) for continuous outcomes (GFR, RBF, ACR, systolic blood pressure, diastolic blood pressure and heart rate), and risk ratio (RR) with 95% CI for dichotomous outcome (death). Heterogeneity is evaluated using the I2 statistic, and I2 > 50% indicates significant heterogeneity^20^. The random-effects model is used for all meta-analysis. We search for potential sources of heterogeneity for significant heterogeneity. Sensitivity analysis is performed to detect the influence of a single study on the overall estimate via omitting one study in turn or performing the subgroup analysis. Owing to the limited number (<10) of included studies, publication bias is not assessed. Results are considered as statistically significant for P <0.05. All statistical analyses are performed using Review Manager Version 5.3 (The Cochrane Collaboration, Software Update, Oxford, UK).

## Results

### Literature search, study characteristics and quality assessment

**Figure. 1:**
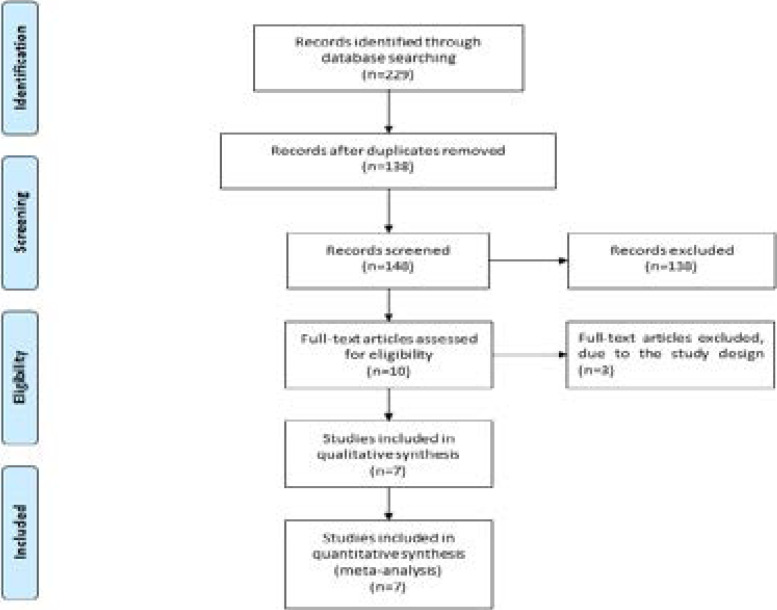
Flow diagram of study searching and selection process.

Figure 1 shows the detail flowchart of the search and selection results. 229 potentially relevant articles are identified initially and 91 duplicates are excluded. Then, 138 papers are removed after checking the titles (n=32)/abstracts (n=106). Three studies are removed because of the study design after reading the full articles, and seven RCTs are finally included in the meta-analysis^12^–^15^, ^21^–^23^.

The baseline characteristics of seven included RCTs are shown in Table 1. These studies are published between 2016 and 2017, and the total sample size is 9766. Among seven included RCTs, liraglutide is administered at the dose ranging from 0.3 mg/day to 1.8 mg/day. The treatment duration ranges from 12 to 24 weeks.

**Table 1:** Characteristics of included studies

NO.	Author	Liraglutide group						Control group						Jada scores
		Number	Age (years)	Female (n)	Body mass index (kg/m^2^)	Duration of diabetes	Methods	Number	Age (years)	Female (n)	Body mass index (kg/m^2^)	Duration of diabetes	Methods	
1	Von Scholten 2017	27	65±7	5	31.9±5.0	15±7	liraglutide (1.8 mg/d) for 12 weeks	27	65±7	5	31.9±5.0	15±7	placebo	4
2	Mann 2017	4668	64.2	1657	32.5	12.8	liraglutide at 0.6 mg daily for 1 week, 1.2 mg for an additional week, and a potential maximum dosage thereafter of 1.8 mg based on tolerance, as determined by the investigator for 6 months	4672	64.4	1680	32.5	12.9	placebo	5
3	Tonneijck 2016	19	60.5±7.2	5	32.0 (30.9–35.9), median (interquartile range)	7 (4–13), median (interquartile range)	liraglutide (1.8 mg/day) for 12 weeks	17	65.8 6 5.8	4	30.8 (28.9 – 31.5)	8 (5–12)	placebo	4
4	Skov 2016	11	54 ± 5	0	29 ± 3	2.9 ± 1.7	a single dose of 1.2 mg liraglutide daily	11	54 ± 5	0	29 ± 3	2.9 ± 1.7	placebo	3
5	Idorn 2016	10	60.7±3.2	3	30.2±1.3	-	titrated to a maximum dose of 1.8 mg daily for 12 weeks	10	63.1±2.1	2	30.8±1.0	-	placebo	3
6	Davies 2016	140	68.0±8.3	65	33.4±5.4	15.9 (8.9)	a single dose of 1.8 mg liraglutide daily for 26 weeks	137	66.3±8.0	72	34.5±5.4	14.2±7.5	placebo	5
7	Bouchi 2016	8	57±16	3	27.7±2.5	-	Liraglutide administered from 0.3 mg/day, increased to 0.6 mg after one week and 0.9 mg after a further week, up to 24 weeks	9	60±22	6	28.2 ± 2.5	-	placebo	3

Among seven included RCTs, two trials report GFR12, 15, two trials report RBF14, 15, two trials report ACR14, 23, four trials report systolic blood pressure and diastolic blood pressure12, 14, 15, 23, three trials report heart rate12, 14, 15, and two trials report death13, 22. Jadad scores of the seven included studies vary from 3 to 5, and all seven studies have high-quality based on the quality assessment.

### Primary outcomes: GFR and RBF

**Figure. 2:**
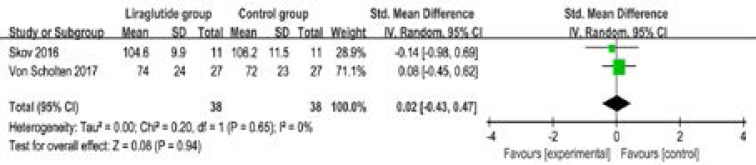
Forest plot for the meta-analysis of GFR.

**Figure. 3:**
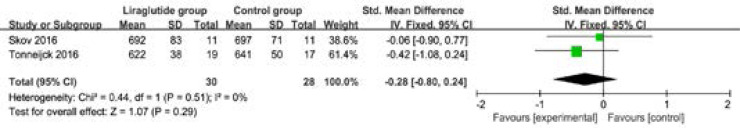
Forest plot for the meta-analysis of RBF.

The random-effect model is used for the analysis of primary outcomes. The results find that compared to control group in type 2 diabetes, liraglutide treatment shows no significant effect on GFR (SMD=0.02; 95% CI=-0.43 to 0.47; P=0.94) with no heterogeneity among the studies (I2=0%, heterogeneity P=0.65, Figure 2), or RBF (SMD=-0.28; 95% CI=-0.80 to 0.24; P=0.29) with no heterogeneity among the studies (I2=0%, heterogeneity P=0.51, Figure 3).

### Sensitivity analysis

There is no heterogeneity for the primary outcome, and thus we do not perform the meta-analysis via omitting one study or subgroup analysis to detect the heterogeneity.

### Secondary outcomes

**Figure. 4:**
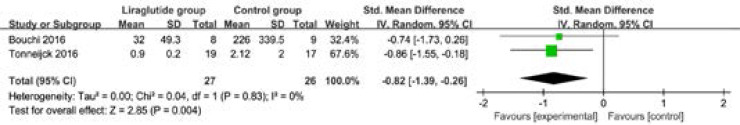
Forest plot for the meta-analysis of ACR.

**Figure. 5:**
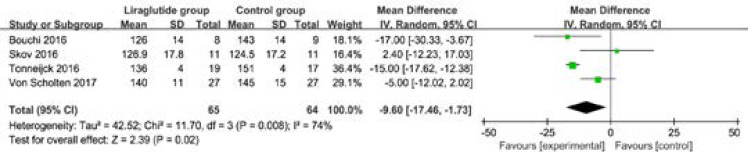
Forest plot for the meta-analysis of systolic blood pressure (mmHg).

**Figure. 6:**
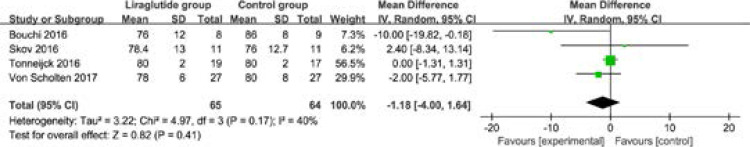
Forest plot for the meta-analysis of diastolic blood pressure (mmHg).

**Figure. 7:**
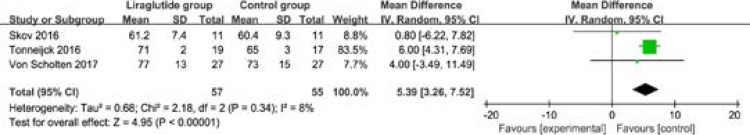
Forest plot for the meta-analysis of heart rate (bpm).

**Figure. 8:**
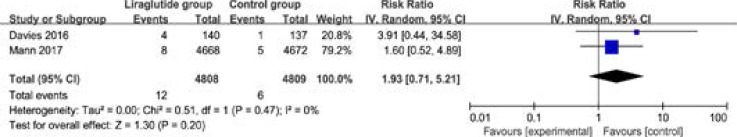
Forest plot for the meta-analysis of death.

In comparison with control intervention in type 2 diabetes, liraglutide treatment is associated with the substantial decrease in ACR (SMD=-0.82; 95% CI=-1.39 to -0.26; P=0.004; Figure 4) and systolic blood pressure (MD=-9.60; 95% CI=-17.46 to -1.73; P=0.02; Figure 5), but has no obvious influence on diastolic blood pressure (MD=-1.18; 95% CI=-4.00 to 1.64; P=0.41; Figure 6). In addition, liraglutide treatment can result in the increase in heart rate (MD=5.39; 95% CI=3.26 to 7.52; P<0.00001; Figure 7) than placebo, but shows no effect on death (RR=1.93; 95% CI=0.71 to 5.21; P=0.20; Figure 8) in patients with type 2 diabetes.

## Discussion

Diabetes has become the most common cause of end-stage renal disease^24^–^28^, and a robust relationship is observed between magnitude of short term albuminuria reduction and long-term slowing of chronic kidney disease progression as well as reduced cardiovascular event rates^29^, ^30^. Short-term albuminuria reduction can lead to long-term renal protection across different interventions and populations, and a 30 % reduction in albuminuria seemed to confer a detectable reno-protective treatment effect^31^.

GLP-1 agonist liraglutide has been widely used for the treatment of type 2 diabetes and lowering HbA 1c32^34^. Reductions in systolic blood pressure, HbA 1c, GFR and body weight may contribute in lowering albuminuria and protecting renal function. In a 12-week, randomized, double-blind trial involving 55 patients with type 2 diabetes, treatment with liraglutide showed no substantial effect on measured renal hemodynamics or renal damage markers of tubular functions or alteration^14^. Our meta-analysis suggests that compared to placebo in type 2 diabetes, liraglutide treatment had no beneficial effect on GFR, RBF or death, but is associated with the decrease in ACR and systolic blood pressure.

One RCT aimed to investigate the effect of liraglutide treatment on renal function in type 2 diabetic patients with persistent albuminuria, and the results found that liraglutide treatment was associated with a statistically and clinically significant reduction in albuminuria. This beneficial effect on albuminuria may be attributed by the reductions in Ang II of 43 % and renin concentrations of 37 % after liraglutide treatment compared with reductions of 28 % and 27 %, respectively, with placebo treatment12. This beneficial effect of liraglutide on albuminuria was also confirmed by another RCT involving 9340 patients with type 2 diabetes and high cardiovascular risk. The new onset of persistent macroalbuminuria occurred in fewer participants in the liraglutide group than in the placebo group (161 vs. 215 patients; hazard ratio, 0.74; 95% CI, 0.60 to 0.91; P=0.004)^13^.

### Limitations

Our analysis is based on only seven RCTs, and more RCTs with large sample size should be conducted to explore this issue. Next, although there is no significant heterogeneity, different doses and treatment duration of liraglutide may produce some bias. Finally, various stages of diabetic kidney disease may have some effect on efficacy evaluation, but it is not feasible to perform their subgroup analysis based on current included RCTs.

## Conclusion

Liraglutide treatment may provide some benefits for the protection of renal function in type 2 diabetes, but more studies should be conducted to explore this issue.
